# Uptake of intermittent preventive treatment of malaria in pregnancy using sulfadoxine-pyrimethamine (IPTp-SP) in Uganda: a national survey

**DOI:** 10.1186/s12936-022-04299-z

**Published:** 2022-10-07

**Authors:** Edward Kwabena Ameyaw

**Affiliations:** Institute of Policy Studies and School of Graduate Studies, Lingnan University, Tuen Mun, Hong Kong

**Keywords:** Malaria, Pregnancy, Public health, Maternal health, Uganda

## Abstract

**Background:**

In spite of the missed opportunities of sulfadoxine-pyrimethamine (IPTp-SP) in Uganda, scanty literature exist on malaria in pregnancy. To date, empirical national study utilizing the 2018-19 Uganda Malaria Indicator Survey to explore predictors of attaining three or more doses of IPTp-SP in the country is non-existent. This study investigated the factors affecting uptake of three or more IPTp-SP doses as recommended by the World Health Organization.

**Methods:**

Data from the 2018–2019 Uganda Malaria Indicator Survey (2018-19 UMIS) was analysed. Adequate uptake of intermittent preventive therapy with IPTp-SP was the dependent variable for this study. Weighted frequencies and percentages were used to present the proportion of women who had adequate IPTp-SP uptake or otherwise with respect to the independent variables. A three-level multilevel logistic regression was fitted. The Bayesian Deviance Information Criterion (DIC) was used in determining the goodness of fit of all the models.

**Results:**

Less than half of the surveyed women had three or more IPTp-SP doses during their last pregnancies (45.3%). Women aged 15–19 had less odds of receiving at least three IPTp-SP doses compared to those aged 45–49 [aOR = 0.42, Crl = 0.33–0.98]. Poor women [aOR = 0.80, Crl = 0.78–0.91] were less likely to have three or more doses of IPTp-SP relative to rich women. Most disadvantaged regions were aligned with less likelihood of three or more IPTp-SP uptake [aOR = 0.59, CI = 0.48–0.78] compared to least disadvantaged regions. The variation in uptake of three or more IPTp-SP doses was substantial at the community level [σ^2^ = 1. 86; Crl = 11.12–2.18] than regional level [σ^2^ = 1.13; Crl = 1.06–1.20]. About 18% and 47% disparity in IPTp-SP uptake are linked to region and community level factors respectively.

**Conclusion:**

IPTp-SP interventions need to reflect broader community and region level factors in order to wane the high malaria prevalence in Uganda. Contextually responsive behavioural change communication interventions are required to invoke women’s passion to achieve the recommended dosage.

## Background

Malaria infection in pregnancy (MiP) is acknowledged as a weighty public health challenge and have ample dangers for the pregnant woman and her fetus [1–3]. The symptoms and complications of MiP fluctuate with respect to intensity of transmission within a defined geographical area as well as a woman’s level of acquired immunity [1, 2]. Nineteen countries within sub-Saharan Africa (SSA), with Uganda inclusive, and one Asian country account for 85% of the global malaria burden [1]. In 2018 alone, about US$ 2.7 billion investment was made into malaria control and elimination globally and three-quarters of this was directed to the World Health Organization (WHO) African Region. In spite of this, malaria continue to take a heavy toll on government and household expenses in Uganda [4]. Pregnant women have increased susceptibility to malaria and its associated complications such as maternal anaemia, stillbirth, low birth weight, and in worse scenarios, infant mortality and morbidity [5, 6]. MiP could be an impediment to the realization of targets 3.1 of the Sustainable Development Goals, thus reducing maternal mortality ratio to less than 70 deaths per 100,000 live births [7].

In order to shield women in moderate to high malaria transmission areas in Africa and their newborns from the adverse implications of MiP and its associated imminent problems, the WHO in 2012 revised its anti-malaria policy and recommended that all pregnant women within such regions should receive at least three doses of intermittent preventive treatment in pregnancy with antimalarial drug sulfadoxine-pyrimethamine (IPTp-SP) [8]. This recommendation was informed by the stagnated IPTp coverage rates and new evidence that reinforced the need for three doses or more [9]. Further, in 2016, the WHO developed new antenatal care (ANC) guidelines by indorsing an increase in the number of ANC to at least eight contacts between pregnant women and healthcare providers as a strategy to enhance prospects of IPTp-SP uptake [2].

IPTp-SP is to be taken by all pregnant women in moderate to high malaria transmission areas and should commence as early as possible within the second trimester. The doses are to be administered at least three times with at least one month interval until childbirth. Generally IPTp-SP declines episodes of MiP, neonatal mortality, low birth weight, and placental parasitaemia [2]. Empirical evidence indicate that IPTp contributes to 29%, 38% and 31% reduction in the incidence of low birth weight, severe malaria anaemia and neonatal mortality respectively [10, 11]. In 2018, out of the 36 African countries that recounted IPTp-SP coverage rates, about 31% of eligible women in the reproductive age received the recommended doses and this signified an increase relative to the rate reported in 2017 (22%) and 2010 (2%). In the case of Uganda, where malaria is endemic in 95% of the country [4], the 2019 World Malaria Report indicated that 30% or lower of the eligible women had three or more IPTp-SP doses [1]. Resistance of the parasite to SP has been noted, however, IPTp still remains a very cost-effective and a promising lifesaving intervention [12, 13].

In spite of the missed IPTp-SP opportunities in Uganda in the wake of high MiP [1, 14, 15], scanty literature exist on MiP in Uganda. The few studies have either been limited to some regions of the country [16–20], used relatively old national data [21], assessed the impact of intermittent preventive treatment during pregnancy [22] and among others. To date, empirical national study utilizing the 2018-19 Uganda Malaria Indicator Survey that explore predictors of attaining three or more doses of IPTp-SP in the country is non-existent. With the aim of invigorating a critical evidence-based discussion on MiP prevention, and offering empirical evidence to guide MiP policies, this study investigated the rate of uptake and factors affecting uptake of three or more IPTp-SP doses as recommended by the WHO.

## Methods

### Data description

Data from the 2018–2019 Malaria Indicator Survey (2018-19 UMIS) of Uganda was analysed. This is a cross-sectional survey that is executed by the Uganda Bureau of Statistics (UBOS) and the National Malaria Control Division (NMCD), however, technical assistance was granted by the Inner City Fund (ICF) [23]. The survey sampled participants through a two-stage sampling design with the intent of achieving estimation of three essential indicators, thus rural-urban locations, all fifteen administrative regions and national coverage. The sampling commenced with selection of clusters from refugee and non-refugee sample frames [23] from the enumeration areas delineated for the 2014 National Population and Housing Census (NPHC). In all, 320 clusters were selected from non-refugee sample frame (236 and 84 from rural and urban settlements respectively). Urban settlements were oversampled to obtain unbiased estimations for rural and urban settlements. The same procedure was followed to select 22 clusters from the refugee frame. The next sampling phase involved the systematic selection of households and 28 households per cluster were selected resulting in a total of 8,878. Eligible women were those aged 15–49 and were either permanent residents or visitors who joined the household the night before the survey. In all, 8,231 women were successfully interviewed signifying 98% and 99% response rates for non-refugee and refugee settlements respectively. In the present study, however, 4,254 women were eligible for inclusion based on completeness of data.

## Measurement of variables

### Dependent variable

Adequate uptake of intermittent preventive therapy using IPTp-SP was the dependent variable for this study. During the 2018-19 UMIS, women were asked if they took IPTp-SP (Fansidar™), and the number of times this was taken during their last pregnancy. The current recommendation by the WHO endorses at least three doses for all pregnant women in locations that have moderate to high malaria transmission of which Africa and for that matter Uganda is inclusive [8, 24]. Following this recommendation, all women who revealed that they had at least three doses of IPTp-SP were categorized as having adequate IPTp-SP (coded 1) whilst those who had less than three were categorized otherwise (coded 0).

### Independent variables

A total of ten (10) independent variables were included and were categorized under individual, community and region-level factors. This was possible due the hierarchical nature of the data set. The individual-level factors comprised age in completed years (15–19, 20–24, 25–29, 30–34, 35–39, 40–44, 45–49) and education measured as highest level of educational attainment (no education, primary, secondary, higher). In addition were wealth quintile (poor, middle, rich), whether mosquito bite causes malaria (yes or no), if sleeping under ITN prevents malaria (yes or no) and if malaria can be prevented by destroying mosquito breeding site (yes or no). The community-level factors comprised residential status (urban, rural, refugee settlement), and socio-economic disadvantage at the community level. The sole region-level factor was socio-economic disadvantage at the region level. Several studies using the DHS dataset have followed the same categorization [21, 25, 26].

### Statistical analyses

Stata version 13 was utilized for all the analyses. Weighted frequencies and percentages were used to present the proportion of women who had adequate IPTp-SP uptake or otherwise with respect to the independent variables (see Table 1). The association of significance between the explanatory variables and adequate IPTp-SP uptake was assessed with chi-square at 5% margin of error. Finally, a three-level multilevel logistic regression was fitted with five models. First of the five models was the empty model without any explanatory variable (Model I). The empty model is an unconditional model, which accounted for the magnitude of variance between community and region levels. This was followed by a model bearing all the individual-level variables (Model II) and subsequently Model III, which accounted for only community-level variables. Model IV featured region-level variables alone whilst the complete and ultimate model included variables of all the aforementioned levels/models (Model V). Output from the models comprised fixed and random effects. The fixed effects were reported as adjusted odds ratios (aORs) at 95% credible intervals (CrIs). However, the random effects reflected as intraclass correlation coefficient (ICC) and median odds ratio (MOR) [27]. With the ICC, it was possible to gauge the extent of variance in the possibility or tendency of adequate IPTp-SP uptake that is explained by community and region level factors. On the order hand, the MOR quantified the community and region variance as odds ratios and in addition estimated the likelihood of adequate IPTp-SP uptake that is influenced by community and region level issues. Groups having least observations were set as reference groups in the models.

**Table 1:** Sample by IPTp-SP utilization during last pregnancy

Variable	At least 3 doses of IPTp-SP in last Pregnancy			
	No n (%)	Yes n (%)	Total n (%)	X ^2^; p-value
Individual level				
Age				19.638; p < 0.05
15–19	143(50.1)	142(49.9)	286(100)	
20–24	553(52.0)	510(48.0)	1063(100)	
25–29	606(55.8)	479(44.2)	1085(100)	
30–34	497(56.4)	385(43.6)	882(100)	
35–39	318(54.8)	262(45.2)	580(100)	
40–44	163(59.6)	110(40.4)	273(100)	
45–49	49(58.2)	35(41.8)	84(100)	
Education				45.638; p < 0.01
No education	379(55.1)	309(44.9)	689(100)	
Primary	1294(55.0)	1060(45.0)	2354(100)	
Secondary	537(54.4)	450(45.6)	987(100)	
Higher	118(52.9)	105(47.1)	224(100)	
Wealth quintile				97.638; p < 0.001
Poor	572(53.8)	491(46.2)	1063(100)	
Middle	863(56.9)	654(43.1)	1517(100)	
Rich	894(53.4)	780(46.6)	1675(100)	
Mosquito bite causes malaria				18.832; p < 0.01
No	1573(57.4)	1166(42.6)	2739(100)	
Yes	755(49.9)	759(50.1)	1515(100)	
Sleeping under ITN prevents malaria				24.221; p < 0.01
No	553(60.0)	368(40.0)	920(100)	
Yes	1776(53.3)	1557(46.7)	3333(100)	
Destroying mosquito breeding site prevents malaria				19.361; p < 0.01
No	1909(54.0)	1624(46.0)	3533(100)	
Yes	420(58.3)	301(41.7)	720(100)	
Community level factors				
Residential status				52.873; p < 0.001
Urban	493(52.4)	448(47.6)	940(100)	
Rural	1664(56.2)	1298(43.8)	2962(100)	
Refugee settlement	172(49.1)	179(50.9)	351(100)	
Zone				45.911; p < 0.001
Southern	719(56.0)	565(44.0)	1284(100)	
Eastern	617(55.6)	493(44.4)	1110(100)	
Northern	465(54.2)	392(45.8)	858(100)	
Western	528(52.7)	474(47.3)	1002(100)	
Socio-economic disadvantage				109.111; p < 0.001
Tertile 1(least disadvantaged)	935(53.2)	822(46.8)	1757(100)	
Tertile 2	855(58.1)	615(41.9)	1470(100)	
Tertile 3(most disadvantaged)	539(52.5)	487(47.5)	1026(100)	
Region level factor				
Socio-economic disadvantage				59.651; p < 0.001
Tertile 1(least disadvantaged)	1048(55.2)	851(44.8)	1898(100)	
Tertile 2	860(54.2)	725(45.8)	1585(100)	
Tertile 3(most disadvantaged)	421(54.7)	349(45.3)	770(100)	
Total	2329(54.7)	1925(45.3)	4254(100)	

Source: 2018-19 Uganda Malaria Indicator Survey

### Model fit and specifications

First, multicollinearity between the explanatory variables was assessed using the Variance Inflation Factor (VIF) [28] and the results showed that the variables were not highly correlated to warrant a concern (mean VIF = 1.55, minimum VIF = 1.19, maximum VIF = 2.09). Second, the Bayesian Deviance Information Criterion (DIC) was used in determining the goodness of fit of all the models. Third, the Markov Chain Monte Carlo (MCMC) estimation was applied in modelling [29] and all models were specified using 3.05 version of MLwinN package in the Stata software.

### Ethics approval

The 2018–2019 UMIS had approval from the Uganda National Council for Science and Technology (UNCST), the Ethics Committee of the School of Medicine Research and Ethics Committee (SOMREC) of the Makerere University as well as the institutional review board of the ICF. The author applied and was granted access to utilize the dataset for the purpose of this study.

## Results

### Descriptive findings

As shown in Table 1, less than half of the surveyed women had three or more IPTp-SP doses during their last pregnancies (45.3%). All the explanatory variables showed significant association at 95% level of significance. Nearly half of women aged 15–19 (49.9%) and the highly educated (47.1%) women received at least three doses. A greater section of rich women had at least three IPTp-SP doses (46.6%) and 50.1% of women who knew that mosquito bite causes malaria did the same. A significant proportion of women who knew that sleeping under ITN prevents malaria (46.7%) and those who did not know that destroying mosquito breeding sites prevents malaria (46.0%) received at three or more doses of IPTp-SP. Receiving at least three doses of IPTp-SP was profound in refugee settlements (50.9%), Western zone (47.3%), most disadvantaged communities (47.5%) and moderately disadvantaged regions (45.8%).

### Fixed effects

Table 2 presents the findings of the fixed effects. The complete and final model (Model V) indicates that women aged 15–19 had less odds of receiving at least three IPTp-SP doses compared to those aged 45–49 [aOR = 0.42, Crl = 0.33–0.98]. Those who had no formal education were less likely to achieve the minimum recommended doses [aOR = 0.51, Crl = 0.35–0.81]. Similarly, poor women [aOR = 0.80, Crl = 0.78–0.91] and women who knew that mosquito bite can cause malaria [aOR = 0.84, Crl = 0.73–0.96] were less likely to have three or more doses of IPTp-SP relative to the rich women and women who did not know that mosquito bite can cause malaria respectively. Women who reported that sleeping under ITN prevents malaria had higher odds of three or more IPTp-SP doses [aOR = 1.22, Crl = 1.04–1.43] relative to those who reported otherwise. The findings further revealed that urban residents were less probable to have at least three doses [aOR = 0.60, CI = 0.46–0.82] as well as most disadvantaged communities [aOR = 0.67, CI = 0.50–0.86] relative to rural residents and least disadvantaged communities correspondingly. Similarly, most disadvantaged regions were aligned with less likelihood of three or more IPTp-SP uptake [aOR = 0.59, CI = 0.48–0.78] compared to the least disadvantaged regions.

**Table 2:** Individual, community and region-level predictors of IPTp-SP utilization

	Model I	Model II		Model III	Model IV	Model V
		aOR [95% Crl]		aOR [95% Crl]	aOR [95% Crl]	aOR [95% Crl]
Fixed effects						
Individual level						
Age						
15–19		0.22** [0.11–0.81]				0.42**[0.33–0.98]
20–24		0.43*[0.24–0.98]				0.38*[0.25–0.79]
25–29		0.99[0.62–1.62]				0.81[0.53–1.22]
30–34		1.01[0.62–1.64]				0.81[0.53–1.24]
35–39		0.94[0.56–1.55]				0.76[0.48–1.18]
40–44		0.99[0.58–1.71]				0.81[0.50–1.30]
45–49		1[1]				1[1]
Education						
No education		0.33**[0.17–0.77]				0.51**[0.35–0.81]
Primary		0.53*[0.44–0.82]				0.62*[0.51–0.91]
Secondary		1.09[0.74–1.60]				1.06[0.75–1.50]
Higher		1[1]				1[1]
Wealth quintile						
Poor		0.81*[0.79–0.94]				0.80**[0.78–0.91]
Middle		0.89[0.75–1.06]				0.88[0.74–1.05]
Rich		1[1]				1[1]
Mosquito bite causes malaria						
No		0.85^*^[0.74–0.97]				0.84^*^[0.73–0.96]
Yes		1[1]				1[1]
Sleeping under ITN prevents malaria						
No		1[1]				1[1]
Yes		1.23^**^[1.06–1.43]				1.22^*^[1.04–1.43]
Destroying mosquito breeding site prevents malaria						
No		1.16[0.97–1.39]				1.165[0.96–1.41]
Yes		1[1]				1[1]
Community level factors						
Residential status						
Urban				0.69***[0.31–0.88]		0.60***[0.46–0.82]
Rural				0.78*[0.68–0.96]		0.80[0.61–1.05]
Refugee settlement				1[1]		1[1]
Zone						
Southern				1[1]		1[1]
Eastern				1.05[0.74–1.49]		1.07[0.71–1.61]
Northern				1.08[0.73–1.61]		1.12[0.68–1.83]
Western				1.19[0.84–1.68]		1.22[0.81–1.84]
Socio-economic disadvantage						
Tertile 1(least disadvantaged)				1[1]		1[1]
Tertile 2				0.87[0.72–1.05]		0.91[0.73–1.12]
Tertile 3(most disadvantaged)				0.71*[0.65–0.92]		0.67**[0.50–0.86]
Region level factor						
Socio-economic disadvantage						
Tertile 1(least disadvantaged)					1[1]	1[1]
Tertile 2					0.71**[0.51–0.89]	0.68***[0.54–0.82]
Tertile 3(most disadvantaged)					0.55**[0.39–0.78]	0.59***[0.48–0.78]
Random effects						
Region level						
Variance (SE)	1.13[1.06–1.20]		1.14[1.04–1.21]	1.12[1.05–1.20]	1.15[1.07–1.21]	1.14[1.06–1.20]
ICC (%)	18.00[15.01–22.90]	17.72[15.20-19.08]		16.33[15.21-9.00]	17.10[16.91–18.70]	18.22[17.90-19.02]
MOR	2.76[2.03–3.42]	2.77[2.65–2.86]		2.74[2.66–2.84]	2.78[2.68–2.86]	2.89[2.33–3.51]
Explained variation	[1]	35.72[29.06–41.20]		31.03[27.61–37.31]	33.20[27.31–37.28]	30.82[26.97–37.91]
Community level						
Variance (SE)	1.86[1.12–2.18]	2.00[1.71–2.31]		1.98[1.14–2.20]	1.59[1.22–1.98]	1.99[1.42–2.36]
ICC (%)	47.60[39.8–50.7]	49.80[37.98–53.70]		48.50[36.90-59.08]	48.40[37.00-51.20]	49.22[38.99–52.51]
MOR	3.67[2.74–4.09]	3.85[3.48–4.26]		3.83[2.77–4.12]	3.33[2.87–3.83]	3.84[3.12–4.33]
Explained variation	[1]	52.00[46.30–59.80]		48.99[41.40-55.81]	47.89[38.21–52.55]	48.70[43.00–52.00]
Model fit statistics						
Bayesian DIC	5839	6002		5998	6010	6012
* N*						
Region level	15	15		15	15	15
Community level	340	340		340	340	340
Individual	4254	4254		4254	4254	4254

^*^ *p* < 0.05, ^**^ *p* < 0.01, ^***^ *p* < 0.001; aOR = adjusted Odds Ratio; CrI = Credible Interval; ICC = Intra-cluster correlation; MOR = Median Odds Ratio; 1 = reference

### Random effects

Outcome of the random effects were also reported in Table 2. The empty model (Model I), revealed that the discrepancy in uptake of three or more IPTp-SP doses was substantial at the community level [σ^2^ = 1. 86; Crl = 11.12–2.18] than regional level [σ^2^ = 1.13; Crl = 1.06–1.20]. Also, the ICC of Model I indicated that 18% and 47% disparity in IPTp-SP uptake are linked to region and community level factors respectively. According to the final model (Model V), the MORs indicate that when a woman changes her community to a community with higher likelihood of three or more doses of IPTp-SP, she has 3.83 higher chances of achieving the recommended doses (i.e. three or more). Similarly, moving to a region with high likelihood of three or more IPTp-SP doses is associated with 2.74-fold increase in having three or more IPTp-SP doses.

## Discussion

The aim of this study was to investigate the predictors of three or more IPTp-SP doses in Uganda as recommended by the WHO [8, 24]. Less than half of the women met the recommended dosage. This is far below the prevalence in other sub-Sahara African countries such as Ghana (63%) [30] but higher than the proportion of women who obtain at least three doses in Mali (36.7%) [31] and Senegal (37.51%) [32]. The prevalence, however, denotes an appreciation from 16% to 18% as reported by earlier studies based on the 2016 Uganda Demographic and Health Survey dataset [21, 33]. The relative increase in optimal IPTp-SP uptake since 2016 is suggestive that the recent anti-malaria and IPTp-SP initiatives are useful. Yet, for Uganda alone to account for 5% of the global malaria burden [1] is not good enough and more public health sensitization, and behavioural change communication interventions should be intensified. Hitherto, IPTp-SP administration has been dependent on ANC attendance, meanwhile, even where ANC attendance is high, as in the case of Malawi (84.0%) sometimes optimal uptake of IPTp-SP is low (24.8%) [34]. Thus, varied interventions such as adopting technological approaches and mobile phone alerts/reminders could prompt pregnant women to achieve the recommended dosage as a way of augmenting the traditional approach of administering the drug during ANC.

The study revealed that women aged 15–19 had less odds of receiving at least three IPTp-SP doses compared to those aged 45–49. Due to stigmatization, cultural and traditional connotations of adolescent pregnancy in Uganda [35], adolescent pregnant women may be less motivated to frequent health facilities, and thereby have an increased likelihood of missing or having lower IPTp-SP uptake. Recent synthesis of evidence on maternal healthcare utilization also noted that adolescents generally have lower maternal healthcare utilization [36]. Perhaps, having a secluded and special care for adolescent pregnant women may motivate their likelihood of visiting the health facility for the doses. In the event that the healthcare provider forgets to administer IPTp-SP, it is common knowledge that not all adolescents will feel comfortable and confident to query the healthcare provider for the drug whilst she is in a queue with her mothers’ age mates and possibly feels apprehended.

Those who had no formal education and poor women were less likely to achieve the minimum recommended doses. Being poor and/or uneducated denotes disempowerment [26] and there is congruence in the literature about the positive impact of empowerment on maternal healthcare utilization [37, 38] and taking charge of one’s holistic wellbeing [39]. This observation points to the need for the Uganda Government and its partner organizations to appreciate that optimising IPTp-SP utilization transcends beyond provision of funds to secure the drugs. Thus, enhancing education opportunities for women and widening their wealth status such that every woman in the reproductive age will be competitive in finding a decent occupation could facilitate uptake of IPTp-SP in the country. Whilst education offers the knowledge for women to appreciate the need for achieving the recommended dosage, enhanced wealth status will offer the financial or economic power required to offset cost which could have hindered them from accessing the recommended dosage.

Women who knew that mosquito bite can cause malaria were less likely to have three or more doses of IPTp-SP, however, those who knew that sleeping under ITN can prevent malaria had higher odds of three or more IPTp-SP doses. All things being equal, women who are knowledgeable about possible routes and preventive strategies of malaria are expected to utilize all available opportunities to protect themselves and their newborns [40]. Arguably, a greater section of the women may be unsure of the information they possess about the causative and preventive routes. The content of ANC messages delivered throughout the trimesters could reflect issues pertaining to malaria causation and preventive strategies in order for women to be conscious and be appreciative of the need to achieve the full dosage. This is very critical on the account that malaria is endemic in the over 95% of the country [4].

The findings further revealed that urban residents were less probable to have at least three doses. Unlike rural settings, urban centres are usually clean with less hideouts for mosquitoes or limited conducive breeding sites for mosquitos. Consequently, there is a high temptation for urban residents to feel that they are less susceptible to malaria even during pregnancy. However, a rural resident may be concerned about being at increased risk of malaria and hence utilize all means possible to achieve the recommended doses. This finding further indicates that availability of health facilities does not necessarily imply healthcare utilization. Thus, several other factors interrelate to determine utilization. This is because health facilities and health personnel are prevalent in urban settings of Uganda relative to the rural locations [41, 42] and all things being equal, urban residents were expected to have increased chances of achieving the recommended IPTp-SP dosage. Health education among urban women through the mass media may be useful to boost IPTp-SP uptake among them.

Most disadvantaged communities and regions were aligned with less likelihood of three or more IPTp-SP uptake. Being a resident of most disadvantaged communities may be indicative of limited access to IPTp-SP outlets such as health facilities and mobile clinics [42]. As a result, these finding was anticipated. This points to the need for urgent appraisal of existing IPTp-SP administration/interventions and prioritization of the possibilities of increasing access among women in most disadvantageous regions and communities. It is by such approaches that malaria burden in Uganda can be reduced and thereby increase the country’s prospects of achieving SDG target 3.1 [7].

## Strengths and limitations

This study emerged from the most recent national malaria survey of Uganda. Due to the sampling procedure and large sample, it is representative of all women aged 15–49 in Uganda. In spite of this strength, the study has some noteworthy limitations. The study adopted a cross-sectional design and as such causal inference is not permissible. Secondly, since the outcome variable was self-reported, under-reporting or over-reporting of optimal IPTp-SP uptake is plausible. Also, since the study was based on pre-existing data without information on health system factors, I was unable to interrogate health system factors.

## Conclusion

The study revealed that less than half of Ugandan women achieved the recommended IPTp-SP dosage at their last pregnancy preceding the 2018-19 UMIS. Community and region level factors are significant predictors of optimal IPTp-SP uptake. All existing IPTp-SP interventions that focus on individual level factors alone need to be reviewed to reflect broader community and region level factors in order to wane the high malaria prevalence in the country. More especially, augmenting IPTp-SP uptake in most disadvantaged communities would require much scrutiny into suitable approaches to ensure access by obviating all barriers. Also, contextually responsive behavioural change communication interventions could invoke women’s passion to achieve the recommended dosage.

## Data Availability

The dataset supporting the conclusions of this article is available in the Measure DHS repository, [https://www.malariasurveys.org].

## Abbreviations

ANC: Antenatal care
aOR: Adjusted Odds Ratio
CI: Credible Interval
DIC: Deviance Information Criterion
ICC: Intra-cluster correlation coefficient
ITN: Insecticide-treated net
IPTp-SP: intermittent preventive treatment in pregnancy with antimalarial drug sulfadoxinepyrimethamine
MiP: Malaria in pregnancy
MOR: Median odds ratio
NMCD: National Malaria Control Division
NPHC: National Population and Housing Census
SOMREC: School of Medicine Research and Ethics Committee
UMIS: Uganda Malaria Indicator Survey of Uganda
UNCST: Uganda National Council for Science and Technology
VIF: Variance inflation factor
WHO: World Health Organization
